# Predicting Drug-Target Interactions Using Drug-Drug Interactions

**DOI:** 10.1371/journal.pone.0080129

**Published:** 2013-11-21

**Authors:** Shinhyuk Kim, Daeyong Jin, Hyunju Lee

**Affiliations:** School of Information and Communications, Gwangju Institute of Science and Technology, Gwangju, South Korea; Università degli Studi di Milano, Italy

## Abstract

Computational methods for predicting drug-target interactions have become important in drug research because they can help to reduce the time, cost, and failure rates for developing new drugs. Recently, with the accumulation of drug-related data sets related to drug side effects and pharmacological data, it has became possible to predict potential drug-target interactions. In this study, we focus on drug-drug interactions (DDI), their adverse effects (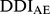) and pharmacological information (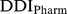), and investigate the relationship among chemical structures, side effects, and DDIs from several data sources. In this study, 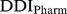 data from the STITCH database, 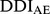 from drugs.com, and drug-target pairs from ChEMBL and SIDER were first collected. Then, by applying two machine learning approaches, a support vector machine (SVM) and a kernel-based L1-norm regularized logistic regression (KL1LR), we showed that DDI is a promising feature in predicting drug-target interactions. Next, the accuracies of predicting drug-target interactions using DDI were compared to those obtained using the chemical structure and side effects based on the SVM and KL1LR approaches, showing that DDI was the data source contributing the most for predicting drug-target interactions.

## Introduction

Computational approaches are promising tools in drug research since they can help reduce the time, costs, and failure rates for developing new drugs. A key problem, however, is that computational approaches have typically been limited in practical applications due to the lack of drug-related data sets, though databases such as DrugBank, KEGG, ChEMBL, and STITCH2 have recently been constructed [1]–[4], thereby enabling opportunities to apply computational approaches to drug research.

To date, several approaches have been proposed for predicting drug-target interactions. Commonly used approaches include docking simulations and literature-based data mining [5], [6]. However, docking simulations cannot be applied to proteins that do not have known 3D structures. Although homology models have been proposed, their accuracies tend to be lower compared to the use of crystal structures, except in a few cases [7]. Another issue is that literature-based data mining methods often rely on the co-occurrence of drugs and proteins in literature and are unable to consider specific drug features, such as its chemical structure [8].

Recently, the relationship between drugs and target proteins has been studied based on aspects such as chemical structure, side effects, drug pharmacology, and protein sequence, with chemical structure-based approaches being the most well-known [9]. The hypothesis of using chemical structures is based on the condition that two molecules having similar chemical structures are likely to target common proteins, and that their chemical structure can then be related to the drug’s effectiveness [10]–[13]; similarly, two proteins having similar sequences are likely to be targeted by the same drugs. Several studies attempting to integrate the chemical structures of drugs and protein sequences have been conducted [14]–[16]. These studies have used different metrics to calculate the similarity between drugs, such as the Tanimoto score and a signature kernel, as well as metrics for similarity between proteins such as the Smith-Waterman alignment of protein sequences and enzyme commission (EC) numbers. In addition, by combining drug and protein similarities, approaches such as the bipartite local model and tensor product of drug and protein kernels have been developed and these integrations have helped to increase the prediction accuracy of drug-target interactions [14]–[16].

Understanding the side effects of drugs is another promising resource. Drugs causing similar side effects are likely to target similar proteins. It was previously found that determining the similarities of drugs based on their common side effects could be highly correlated to the similarities of their chemical structures, and could sometimes be used to predict new drug-target interactions that were not revealed by the similarities of their chemical structures [17]. However, this approach can only be applied to drugs with known side effects. The drug’s pharmacological data is also a useful resource; one study investigated the relationship between the chemical space and the pharmacological space containing their pharmaceutical effects, adverse effects, cautions, usages, properties, etc. [18]. The study showed that determining the pharmacological space is useful for predicting drug-target interactions.

Since the study by Yamanishi *et al.* [18] confirmed that pharmacological spaces are useful in predicting drug-target interactions, we further investigated this concept with respect to drug-drug interactions (DDIs); i.e, how the positive or negative association of two drugs can be used to infer the drug’s target proteins. Note that the process of developing and repositioning drugs is rather complicated because of problems incurred by unexpected effects, such as adverse effects between DDIs, similar activities, and pharmacological actions when drugs are co-administered. Predicting drug-target interactions using their chemical structure and known side effects cannot effectively model these relationships. Nevertheless, integrating multiple features of drugs might be a powerful prediction tool since it focuses on the multiple aspects of drugs–this is why many studies have attempted to integrate multiple features in bioinformatics problems, such as for protein function prediction [19]–[21].

In this study, we investigate the contribution of DDI in the prediction of drug-target interactions. For this task, we first collect two sets of DDIs from known databases. For example, an adverse DDI effect (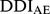) from drugs.com is a modification of the effect of drugs when other drugs are co-administered, consequently leading to severe side effects [22]. In addition, the pharmacological DDI (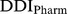) collected from STITCH [4] is a relation between compounds that is derived from similar activities and has similar effects or associations.

The accuracy of predicting drug-target interactions using DDI depends on several factors. First, if we assume that two similar drugs are likely to target the same proteins, the measure of similarity between the two drugs based on DDI should affect the prediction accuracy. Second, there are several drug-target interaction databases; however, since the prediction accuracy depends on gold-standard interactions, it is important to select and compare drug-target interactions from several drug-related databases. For the first issue, we calculated the similarity for DDI based on three measures: direct interactions, the shortest path, and using a diffusion kernel. Each measure was then compared by predicting drug-target interactions using support vector machine (SVM) and kernel-based L1-norm regularized logistic regression (KL1LR) methods. In previous studies, SVM has been widely used for predicting drug-target interactions [14]–[16]. Although KL1LR was not previously applied to the prediction of drug-target interactions, it has proven to be powerful when used for protein function predictions [21]. It should also be noted that although the SVM model gives a high prediction accuracy for drug-target interactions, it is not clear how to use it to study the factors contributing to a drug’s target protein; in contrast, since the KL1LR approach is model-based, it can more easily be used to explore the contribution of other drugs having a high similarity in predicting target proteins. Furthermore, the L1-norm regularization property of KL1LR can generate an explainable model for significant features by assigning some coefficient values to zero [21]. Also, due to its simplicity, its computational time is faster than the SVM approach. For the second issue, we collected two sets of drug-target interactions: ChEMBL [3] and STITCH [4]; we subsequently showed that the prediction results obtained from these two data sets are consistent.

We also compared the contribution of DDI to both the chemical structure and side effect similarities based on the SVM and KL1LR approaches, showing that DDI was the data source contributing the most for predicting drug-target interactions. Next, we integrated the chemical structure, side effect, and DDI data sets to predict drug-target interactions.

Even though this study primarily focuses on DDI, as a final step we further investigated the integration of protein similarity to drug similarity using DDI and then compared our approach to other methods.

## Materials

### DDI Data

In this study, we focused on two DDI data sets: 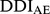 and 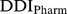. First, we extracted 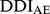 from drugs.com, a website dealing with information pertaining to the adverse and side effects of two drugs [23]. However, data access for drugs.com is not convenient because the information is comprised of unstructured text. Therefore, we had to manually retrieve drug names and 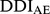 from documents by matching drug synonyms to ChEMBL data [3]. Second, 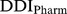 was extracted from STITCH; in STITCH, interactions between chemicals were collected from various resources including similar activity profiles in the anticancer drug screen data of 60 human tumor cell lines (NCI60), pharmacological actions obtained from Medical Subject Headings (MeSH), literature by using natural language processing, and pathway and experimental databases [4].

Although 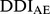 contains the adverse effects of two drugs and 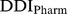 mainly contains two drugs with similar activities or associations, these two data sets were not mutually exclusive. Indeed, it was found that the ratio of common drug interactions between the two DDI data sets was around 10%, based on interactions among the drugs used in this research.

### Drug-target Interaction Data with Chemical Structures and Side Effects

**Figure 1:**
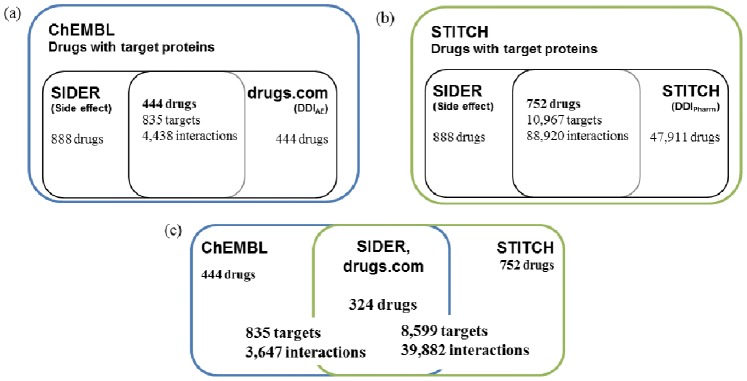
Numbers of drugs, proteins, and drug-target interactions used in this research. (a) Drug-target interactions from ChEMBL are combined with side effects and 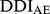 from SIDER and drufgs.com databases. (b) Drug-target interactions from STITCH are combined with side effects and 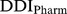 from SIDER and STITCH databases. (c) Common drugs from ChEMBL and STICH are shown with the numbers of target interactions.

Drug-related data sets were extracted from four databases: ChEMBL [3], SIDER [24], STITCH2 [4], and drugs.com [23] (Table 1). Using these databases, we constructed two independent sets of drug-target interactions that were then used as positive gold-standard data sets. For the first data set, we used the ChEMBL database. ChEMBL contains 257,867 compounds with target protein information, 2,733 targets, and 584,516 interactions (version 5). SIDER contains 888 drugs, 1,450 side effects, and 62,269 drug side-effect pairs. Drugs.com is an online drug information resource that includes 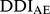; it contains 559 drugs with matched identifiers from ChEMBL drugs, and among them 444 have 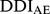 pairs. Since ChEMBL does not contain side effect information and 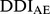 data sets, we determined the possible side effects and 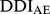 for drugs by matching synonyms of the drugs in ChEMBL, SIDER, and drugs.com. As a result, we found 444 drugs in common, 835 target proteins, and 4,438 drug-target interactions (Figure 1a).

**Table 1:** Drug-related data sets used in this study.

	ChEMBL	SIDER	STITCH	drugs.com
Drug	257,867	888	55,503	559^1^
Protein	2,733	–	14,732	–
Drug-Protein	584,516	–	897,803	–
SE	–	1,450	–	–
Drug-SE	–	62,269	–	–
DDI_AE_ or DDI_Pharm_	–	–	47,911	444^2^

Row names represent the following data: ‘Drug’ is # of drugs with target interactions, Protein’ is # of proteins from humans, ‘Drug-Protein’ is # of drug-target pairs, ‘SE’ is # of side effects, ‘Drug-SE’ is # of drug-side effect pairs, and ‘
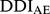
’ or ‘
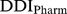
’ is # of drugs having DDI. The two superscripts in the last column represent the following: 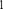 is # of drugs with matched identifiers from ChEMBL drugs with target interactions and SIDER side effects, and 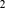 is # of drugs having DDI in 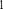.

For the second data set, we extracted drug-target interactions from STITCH, which is derived from NCI60, PubChem, Medical Subject Headings (MeSH) pharmacological actions, and literature using natural language processing (NLP) [4]. STITCH contains 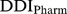 data and we can directly use the side effects in SIDER since STITCH and SIDER use the same drug identifiers. The number of proteins in STITCH is larger than the number of ChEMBL proteins when we combine common drugs from all data sources; 752 drugs and 10,967 proteins, and 88,920 drug-target interactions were obtained (Figure 1b). Note that we only used proteins from Homo sapiens.

Finally, 324 drugs were found to be common between ChEMBL and STITCH (Figure 1c). Here, we mapped common drugs by calculating the chemical structure similarity because the drug identifiers from the two data resources are different. When the similarity between two drugs is 1.0, the two drugs are considered equal. All 324 common drugs are FDA approved drugs, although ChEMBL and STITCH contain compounds as well as FDA approved drugs in their original sets. In these 324 drugs, there were 3,647 and 39,882 drug-target interactions in ChEMBL and STITCH, respectively. These known interactions were then used to estimate the prediction accuracy of the proposed methods and contributions of drug-related data sources for inferring drug-target interactions.

## Methods

**Figure 2:**
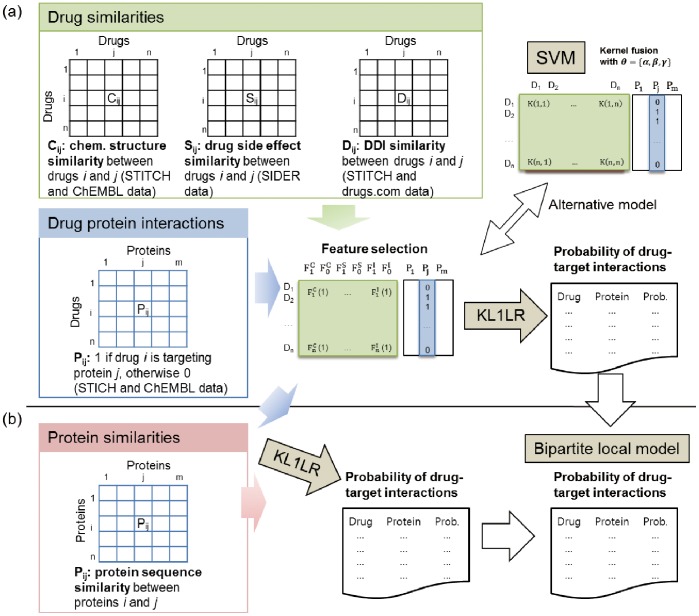
Overview of the entire method procedure. (a) Drug similarities and drug-protein interactions are used to calculate the probabilities of unknown drug-target interactions. Three different drug similarities (chemical structure, drug side effect, and DDI similarity) are applied. Two learning models (KL1IR and SVM) are used to train and test interactions. (b) Protein similarities are integrated with drug similarities to predict unknown drug-target interactions. In this process, the bipartite local model is used.

To predict the drug-target interactions, the similarity between drugs, the similarity between target proteins, or a combination of both similarities can be used. In this section, we first introduce similarity measures between drugs using data sets such as chemical structure, side effects, and DDI. Next, we describe how two classification methods, KL1LR and SVM, are used in predicting drug-target interactions by integrating similarity measures. Then, methods for measuring the similarity between proteins are provided. Finally, approaches for predicting the drug-target interactions using both the similarities between drugs and similarities between proteins are explained. Note that since the main focus of this study is the DDI contribution, the similarity measures between drugs based on DDI and their subsequent integration with other similarities between drugs using KL1LR are extensively explained. Figure 2 depicts the diagram of the entire procedure.

### Drug Similarity Measures

#### Chemical structure-based drug similarity

We obtained the chemical structures of drugs from ChEMBL and STITCH. ChEMBL has several formats for representing chemical structures, such as canonical smiles, mole files, and InChI keys. Among them, we used the canonical smiles format to represent the chemical structure and used an open source library, a small molecule subgraph detector (SMSD) toolkit, to calculate the similarity score of the structures. In SMSD, the Tanimoto similarity was used to detect a maximum common subgraph [25]. Finally, we constructed a similarity score matrix 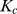 for all drug pairs.

For comparative purposes, the signature kernel between two chemical structures is computed as a dot product between the molecular signature vectors for a given height 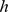, which is a predefined distance from a given atom; the similarity between two chemical structures is 
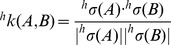
, where 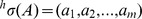, 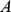 is the chemical structure, 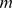 is the number of possible atomic signatures of height 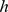, and 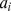 is the presence or absence of the particular atomic signature for height 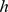 [16]. To implement the signature molecular descriptor, software from [26] was used. The signature kernel matrix is then denoted as 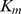, though for convenience, the chemical structure similarity in this study is the Tanimoto score, unless the specific kernel is mentioned.

#### Side effect-based drug similarity

We computed the similarity between drugs based on their side effects, which is computed based on the number of common side effects between them. Here, we employed a down-weighting method to penalize the correlating side effects and the frequently occurring side effects [17], [27]. Correlations between side effects are measured using hierarchical clustering; if two side effects are reported in similar set of drugs, they are clustered together since their correlation is considered high. When a side effect 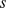 belongs to a large cluster because it is co-related with many other side effects, the weight 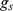 of side effect 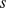 decreases. Here, the frequency penalty weight 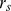 is defined as 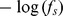, where 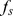 is the frequency of side effect 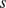 [17]. Finally, the similarity score of two drugs 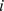 and 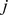 is calculated as follows; let 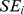 be a set of side effects of drug 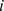, then.
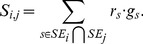
(1)


The similarity 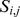 is used as the kernel matrix 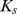 of side effects.

#### DDI-based drug similarity

We computed the DDI similarity using direct interactions, the shortest path, and a diffusion kernel. First, when direct interactions are used, the similarity 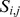 between two drugs 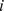 and 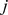 is defined as.
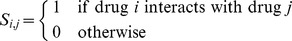
(2)


Second, determination of the shortest path is based on the drug network. For this task, each drug is considered as a node and interactions between the drugs are edges. The similarity 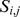 between two drugs 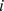 and 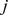 is then defined as 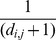, where 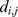 is the shortest distance between the two drugs; this method considers the relationship between drugs even though they do not directly interact. Third, the similarity between drugs using a diffusion kernel is defined as 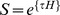, in which 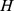 is defined as [28].
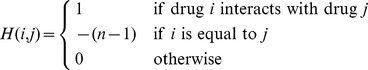
(3)where 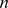 is the number of drugs, 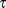 is the diffusion constant, and 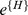 is the matrix exponential of matrix 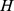. In this case, the diffusion kernel considers both indirect and direct interactions, and controls the contributions of indirect interactions using the diffusion constant 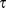. Hence, the similarity 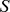 will be used as the kernel 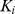 for the DDIs in this paper.

### Predicting Drug-target Interactions Based on Drug Similarities

#### KL1LR model

Previously, KL1LR has been successfully used to predict protein functions [21]; here, we applied KL1LR to predict drug-target interactions. To make this paper self-contained, let us first describe the KL1LR method and then explain how KL1LR was applied to this problem. In brief, for a given protein, let 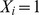 if drug 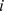 interacts with the protein; otherwise 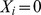. For a given kernel, two feature vectors 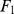 and 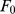 are constructed, where 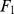 is the average of kernel values between drug 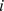 and other drugs that target the given protein, and 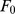 is the average of kernel values between drug 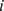 and drugs that do not target the given protein. These features reflect our assumption that the value of 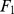 will be high if a drug 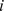 targets the given protein since other drugs with high drugs similarities also target the given protein. Then, a logistic regression model can be used to calculate the corresponding coefficients 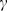, 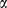, and 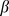, i.e,
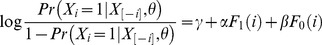
(4)where 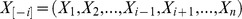, 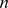 is the number of drugs, and



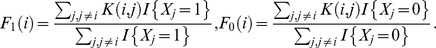
where 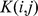 is a kernel value (i.e. a similarity between drug 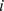 and 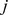) and 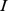 is an indicator function to check whether 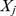 interacts with the given protein. We used an L1-norm regularization to combine multiple data sets. The L1-norm regularization generates an explainable model for multiple features by shrinking non-significant coefficients to zero [29]. Here, Equation (4) can be extended to Equation (5) to combine multiple data sets. For this task, let coefficients 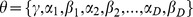, then
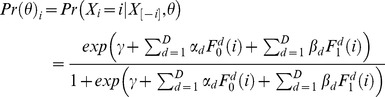
(5)where 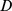 is the number of data sets, and 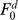 and 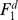 represent 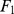 and 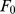 for the 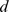-th data set. The log likelihood function from the observed data is subsequently given by



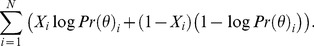
(6)Next, the coefficients 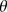 are estimated by maximizing the log likelihood and penalizing coefficients for related features.
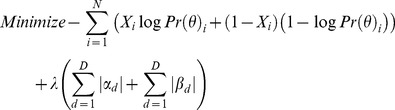
(7)where a regularization parameter 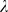 controls the cardinality of 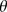. For implementation, we used an interior-point method for KL1LR [30].

#### SVM model

In SVM, drug data sets are represented by kernels, where an element of the kernel is the similarity between two drugs. The kernel matrix of three data sets–chemical structure, side effect, and DDI–will be referred to as 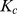, 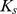, and 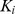, respectively. These kernels are used in SVM to predict drug-target interactions.

SVM can handle both linear and nonlinear data to represent biological data using a *kernel trick* [31]. SVM requires a positive semi-definite kernel. For the case that the kernel matrix is not positive semi-definite, we transform it by adding a small multiple of the identity matrix to the diagonal until all eigenvalues become non-negative [14]. In SVM, a cost parameter 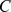 controls the trade-off between the misclassification of training data and the margin that is defined to be the smallest distance between the decision boundary and the training data.

To integrate these three data sets, we generated a combined kernel 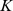 by adding three kernels: 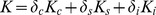, where 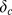, 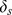, and 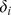 are the kernel fusion coefficients used to give weights.

### Parameters in Prediction Models

Since the prediction accuracy depends on the choice of parameters in a diffusion kernel, KL1LR, and SVM, several values for the following parameters were used in each training and testing procedure and the accuracies were compared in the Results section. These parameters include:

- a diffusion constant 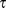 in diffusion kernel,
- a regularization parameter 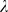 in KL1LR,
- a cost parameter 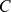 in SVM, and
- kernel fusion coefficients of 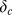, 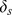, and 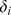 in SVM. For the optimal choice of kernel function coefficients, we tested several combinations of the three coefficients as well as the approach used in a previous study by Lanckriet *et al.* [19], which was successfully applied in a protein-function prediction approach to integrate multiple kernels. To implement the kernel fusion method, software from [32] was used.

In this experiment, several values for KL1LR and SVM parameters were tested as the basis for a rigorous comparison. For KL1LR, 13 different 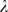 values in [0.01, 0.3] were used; for SVM, 18 different 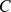 values in [0.1, 100] were tested (accuracies using all these values are shown in Table S4).

### Protein Similarity Measures

The similarity score between two target proteins can be measured based on the protein sequences or enzyme commission (EC) numbers. Amino acid sequences of two proteins are aligned using the Smith-Waterman alignment method [33], with the normalized score used as the similarity measure. The hierarchy score of the enzyme EC number is another method for measuring the similarity [15]. In this method, enzymes are organized into hierarchies that represent specific functions within each family. Hierarchy scores are then computed as the number of common ancestors in the corresponding hierarchy plus one. Two kernel matrices based on the sequences and hierarchies of the EC numbers are denoted as 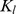 and 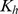, respectively. These protein similarities can also be used to predict drug-target interactions using KL1LR and SVM; thus, for a given drug, the probabilities of targeting proteins can be calculated based on protein similarities.

### Predicting Drug-target Interactions by Integrating Drug And Protein Similarities

To integrate kernels obtained from drugs and proteins, a bipartite local model is used. The model introduced by Bleakley *et al.* [14] calculates the probability of targeting proteins for a given drug using the protein kernel, and also calculates the probability of being targeted by drugs for a given protein using the drug kernel. When these probabilities are calculated for all drugs and proteins, two independent probabilities for the interaction between drugs and proteins are generated for all drug and protein pairs. To aggregate these two probabilities, 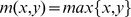 is then used, where 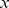 and 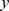 are the probabilities predicted based on the drug and protein kernels. This bipartite local model was applied to both KL1LR and SVM.

For comparative purposes, the tensor product of the drug and protein kernels is also used in this study. This method obtains the inner product between tensor products using 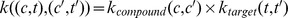, where 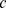 denotes a compound, 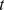 denotes a target protein, and 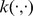 is a kernel value. In two previous studies [15], [16], the tensor product of the two kernels was used as a kernel in SVM.

### Performance Measurement

To assess the prediction performances, we used a 5-fold cross validation. In brief, for a given target protein, drugs are randomly divided into five groups, with the corresponding kernel matrix for the drugs and drug-target pairs also divided into five groups. In each case, four groups are used for training and one is the test group. Next, the probabilities of interactions are calculated for the drug-target pairs in the test group; this process is repeated five times using a different test set each time, and the random division process into five groups is also repeated five times. This 5
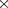
5 fold cross validation was performed for all proteins. Finally, a global accuracy is computed using the area under the ROC curve (AUC) based on the sensitivity (SN) and false positive rate (FPR) values; the interaction probabilities between each drug and target pairs are used to calculate the SN and FPR for different thresholds. The final AUC value is the average of all 25 cases.

## Results

### Predicting Drug-target Interactions based on DDI Similarity

#### Comparison of DDI data

**Figure 3:**
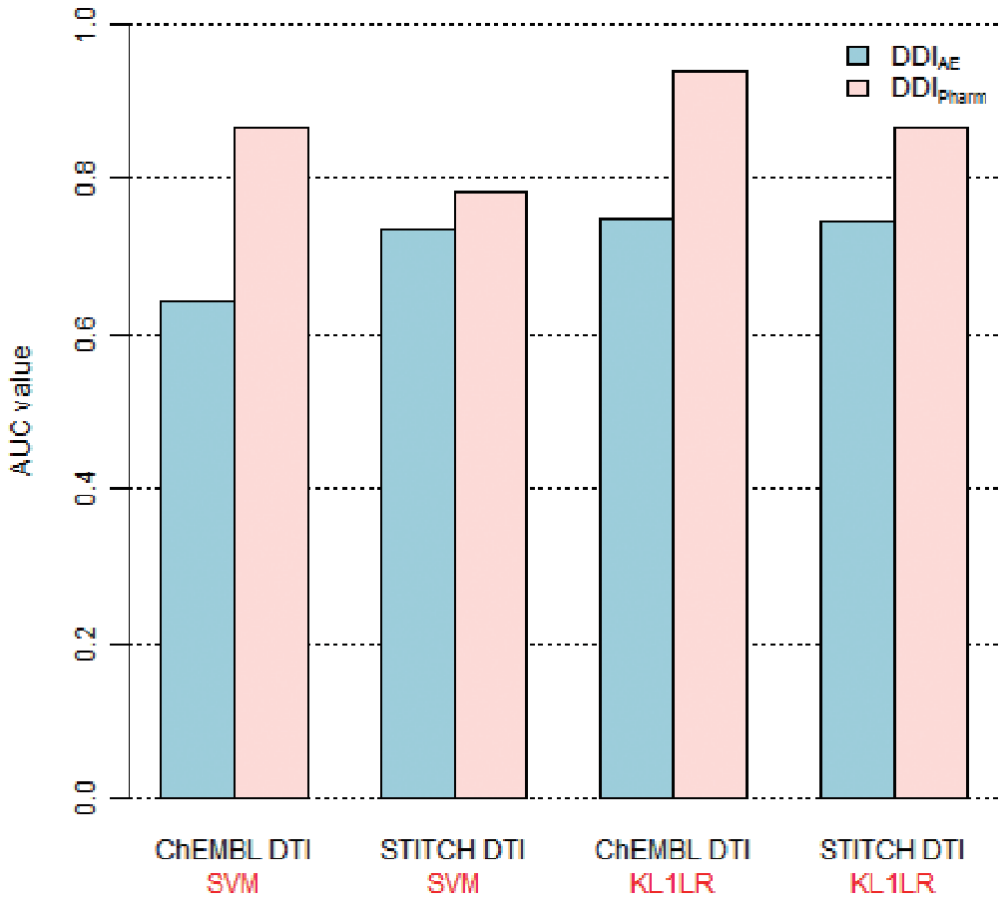
Comparison of STITCH *DDI_Pharm_* and drugs.com. *DDI_AE_*. Two DDI data sets were used to predict ChEMBL and STITCH drug-target interactions (DTI). Each kernel was measured using the shortest path method. 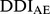 contains 11 unreachable drugs. Therefore, we used 313 drugs from the ChEMBL and STITCH data sets.

We compared two DDI data sets, 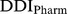 and 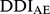, to predict drug-target interactions. In this experiment, we used the shortest path similarity to compute the kernel matrix for the SVM and KL1LR methods. Figure 3 shows the prediction accuracy. Accuracies from 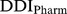 are seen to be consistently higher than those from 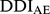 for both ChEMBL and STITCH drug-target interaction data sets and for both SVM and KL1LR methods. This result implies that 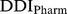 is more useful in predicting drug-target interactions than 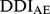. Therefore, in subsequent analyses, we used 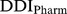 data to represent the DDI.

We further validated the reliability of 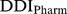 by comparing it with the KEGG pharmacological data set [18]. The KEGG pharmacological information was obtained from package inserts of drugs and its effectiveness in predicting drug-target interactions has been shown in several studies [18], [34], although it only covered 252 drugs. The drugs from KEGG were then classified into four categories: enzyme, GPCR, ion channel, and nuclear receptor. Since the numbers of common drugs with STITCH in the other three categories were small, we used enzyme drugs for comparison. We found 443 common drugs by matching the names of drugs between 212 KEGG enzyme drugs and 49,924 STITCH compounds; the numbers of corresponding proteins were 3,330 and 478, respectively. Then, we measured the accuracies using KL1LR and SVM under the same experimental conditions. Table 2 shows that accuracies using 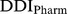 were on average similar to those using KEGG pharmacological information and better for STITCH data set. The benefit of using 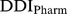 lies in that drugs with 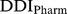 information is larger than those with KEGG pharmacological data with similar accuracies.

**Table 2:** Comparison of 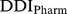 and KEGG pharmacological information.

Methods	Data Sources	Kernels	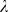 or C	AUC
KL1LR	STITCH	DDI_Pharm_	0.04	0.7508
KL1LR	STITCH	Pharmacology	0.04	0.7369
KL1LR	KEGG	DDI_Pharm_	0.04	0.7316
KL1LR	KEGG	Pharmacology	0.03	0.7232
SVM	STITCH	DDI_Pharm_	2	0.7982
SVM	STITCH	Pharmacology	0.75	0.7685
SVM	KEGG	DDI_Pharm_	30	0.6727
SVM	KEGG	Pharmacology	20	0.7089

Two sets of drug-target interactions from STITCH and KEGG were used for prediction. 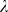 and 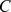 are the KL1LR and SVM parameters, respectively; Table S1 contains prediction results when different parameter values were used. Table S2 contains ROC curves of true positive rate and false positive rate, and tables of true positive, false positive, true negative, and false negative values for each threshold.

### Relative Performance of DDI Similarity Measures

**Figure 4:**
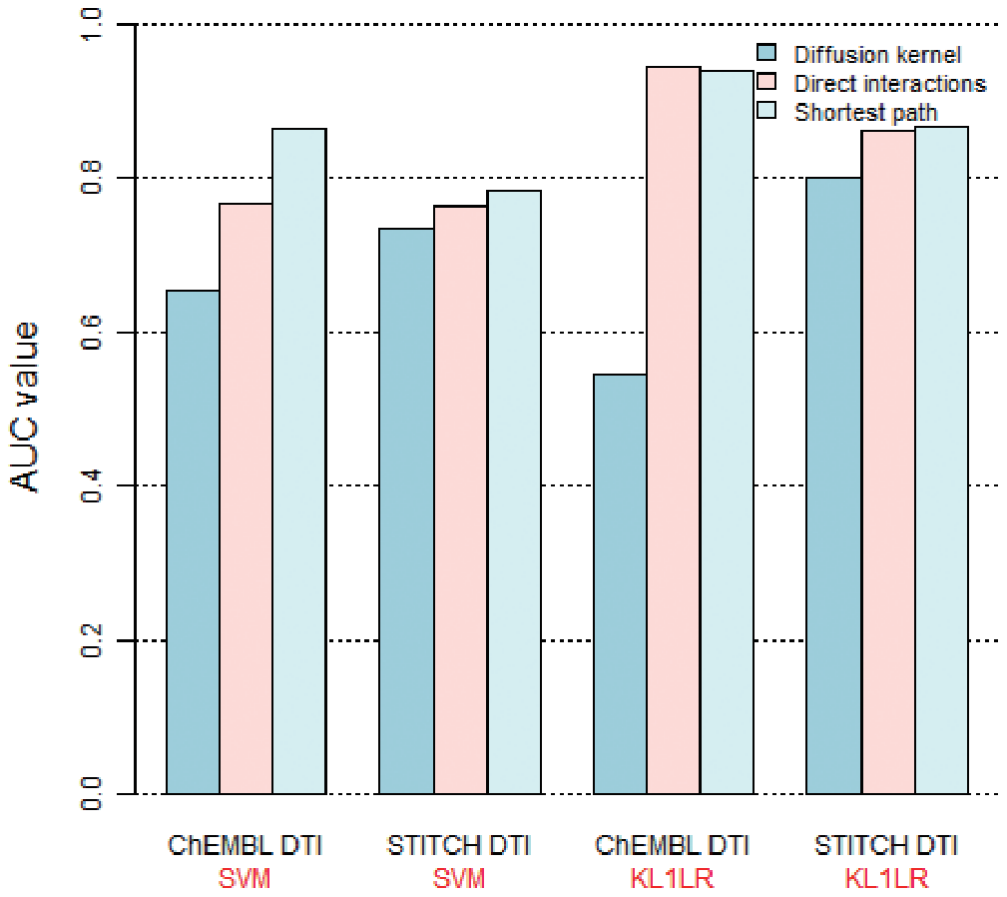
Prediction accuracies of 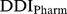 constructed from three similarity measures. Three different measures were used to construct kernels for SVM and KL1LR. These measures were then used to predict ChEMBL and STITCH drug-target interactions.

Three measures were used to calculate the similarity of 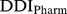: direct interactions, the shortest path, and using a diffusion kernel. Figure 4 presents the prediction accuracy of drug-target interactions depending on these three measures when 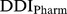 was used. For the diffusion kernel, diffusion constant 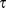 = 0.5 was used after trying different values of 0.1, 0.3, 0.5, and 1.0. Prediction was performed using both SVM and KL1LR approaches. For both ChEMBL and STITCH drug-target interaction data sets, the accuracy using the shortest path was found to be the highest. Therefore, we used the shortest path kernel to measure the similarity between two drugs based on 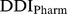 in the following experiments.

### Unknown Drug-target Interactions with High Probabilities

Since the ChEMBL and STITCH data sets might not contain all true drug-target interactions, highly ranked unknown interactions with a high probability from KL1LR might be potential drug-target interaction candidates. Hence, we checked whether these unknown interactions were included in other databases. Among 50 highly ranked unknown interactions in ChEMBL, 13 interactions were found in databases such as STITCH [4], DrugBank [1], KEGG [2], CTD [35], and BindingDB [36], as shown in Table 3. For the purpose of comparison, we also checked whether unknown interactions were found in other databases when the chemical structure similarity was used. Although some unknown interactions were found, the chemical structure similarity predicted several drug-target pairs for the same protein family. For example, Midazolam interacts with GABRA1, GABRA2, GABRA3, GABRA5, and GABRA6. It is also worth noting that two different drug similarities predict different subsets of interaction pairs. All unknown interactions within the top 50 pairs from the ChEMBL and STITCH data sets are listed in Table S5.

**Table 3:** List of highly ranked unknown drug-protein pairs in ChEMBL.

Drug similarity	Drugs	Proteins	Prob.	Evidence
DDI_Pharm_	Nifedipine	SLC22A1	0.678445	CTD
	Verapamil	ADRB1	0.672712	CTD
	Phenytoin	CYP3A4	0.656528	STITCH
	Diphenhydramine	SLC22A1	0.632571	DrugBank
	Amitriptyline	SLC6A2	0.576068	DrugBank
	Midazolam	SLC22A1	0.569028	DrugBank
	Valium	ERG1	0.521953	CTD
	Nifedipine	ADRB1	0.513618	CTD
	Phenytoin	ABCB1	0.503147	STITCH
	Midazolam	GABRA1	0.498508	STITCH
	Fenoprofen	PTGS1	0.494015	DrugBank
	Oxaprozin	PTGS1	0.494015	STITCH
	Metoprolol	HTR1A	0.478448	STITCH
Chem. struc.	Amoxicillin	SLC15A1	0.644264	STITCH
	Midazolam	GABRA5	0.614966	DrugBank
	Midazolam	GABRA3	0.614966	DrugBank
	Midazolam	GABRA2	0.556632	DrugBank
	Midazolam	GABRA1	0.503353	STITCH
	Midazolam	GABRA6	0.414876	KEGG
	Alprazolam	GABRA6	0.408056	DrugBank
	Aspirin	ADRB2	0.339491	CTD
	Rabeprazole	ATP4A	0.33869	STITCH
	Rabeprazole	ATP4B	0.33869	STITCH
	Terbutaline	ADRB1	0.315024	CTD

Among all 270,540 drug-protein pairs from the ChEMBL data set, the top 50 unknown pairs determined by the KL1LR method using 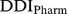 data sets were checked, and the unknown pair was listed if it was found in the STITCH [4], DrugBank [1], KEGG [2], BindingDB [36], and CTD [35] data sets. Drugs in the second column and proteins in the third column are likely to interact, based on the probabilities shown in the fourth column. If interactions are found in more than two data sets, only one source is listed. Similarly, the results obtained using chemical structure similarities are shown.

**Figure 5:**
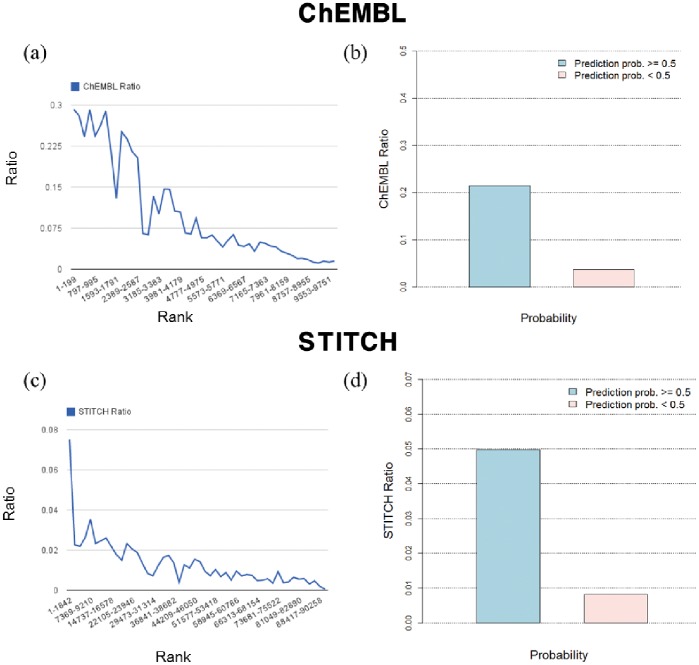
Prediction accuracies of 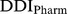 constructed from three similarity measures. Three different measures were used to construct kernels for SVM and KL1LR. These measures were then used to predict ChEMBL and STITCH drug-target interactions. (a) In the different ranges of ranks assigned by probabilities of interactions between drugs and targets in ChEMBL, ratios of known drug-target interactions in STITCH among unknown interactions in ChEMBL are presented according to their ranks. (b) The left bar is the ratio for interactions having prediction probabilities ≥0.5; among 28 unknown interactions in ChEMBL, 6 are known in STITCH. The right bar shows interactions with prediction probabilities <0.5; among 68,334 unknown interactions in ChEMBL, only 2,543 are known in STITCH. (c) In the different ranges of ranks assigned by probabilities of interactions between drugs and targets in STITCH, ratios of known drug-target interactions in ChEMBL among unknown interactions in STITCH are presented according to their ranks. (d) The left bar is the ratio for interactions having prediction probabilities ≥0.5; among 402 unknown interactions in STITCH, 20 are known in ChEMBL. The bar on the right shows interactions with prediction probabilities <0.5; among 88,028 unknown interactions in STITCH, only 712 are known in ChEMBL.

Since Table 3 and Table S3 show that a number of highly ranked unknown drug-target interactions in ChEMBL or STITCH are also found in other data sets, we next checked the ratio of interactions that are unknown in one data set but are included in the other data sets–for a various range of ranks. We posit that highly ranked unknown drug-target pairs are more likely to be included in other data sets if the prediction method performs well; i.e., the ratio should increase when the rank increases. For this task, we first collected 460 common proteins from the two data sets to construct common drug-protein pairs. Then, we calculated the probabilities of all drug-target pairs using the KL1LR method with the 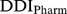 similarity and ranked them. Figure 5a shows the ratios of known drug-target interactions in STITCH among the unknown interactions in ChEMBL; the ratios decrease when the ranks of interactions in ChEMBL decrease. Figure 5b compares the ratios for a threshold of 0.5, where it becomes clear that highly ranked pairs are included in the other data set. Figure 5c and Figure 5d are plots for the STITCH data set. Because the number of interactions in ChEMBL is small compared to all possible drug-target pairs in STITCH, the ratios in Figure 5c and Figure 5d are relatively small compared to Figure 5a and Figure 5b. However, it is similarly observed that ratios in the highly ranked pairs are higher than those in the low ranked pairs. Therefore, these results indicate that highly ranked drug-target pairs are more likely to be true interactions than the lower ranked drug-target pairs.

### Predicting Drug-target Interactions based on DDI, Chemical Structure, and Side Effect Similarities

#### Relationship between DDI similarity with chemical structure and side effect similarities

**Figure 6:**
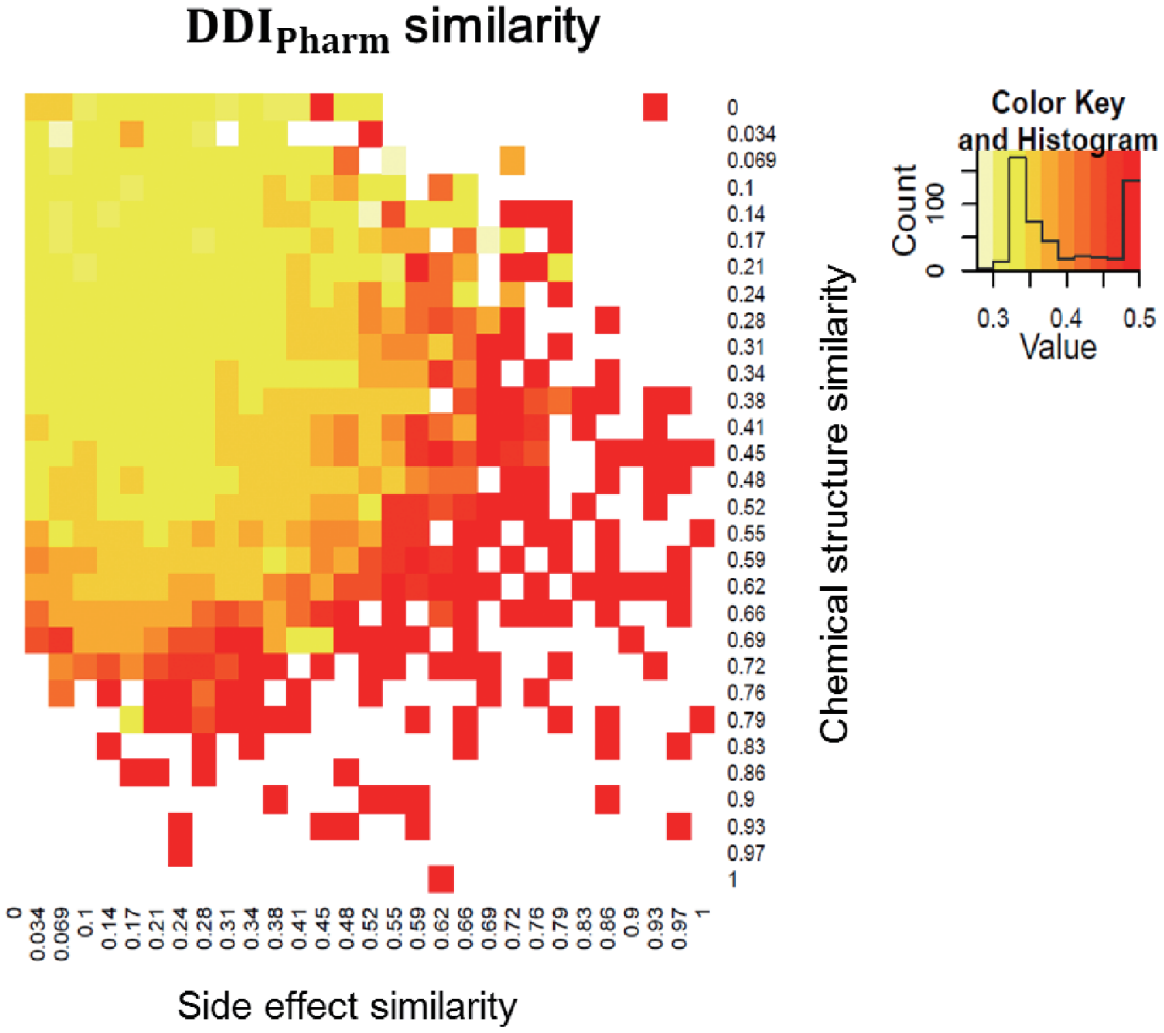
DDIPharm similarity depending on chemical structure and side effect. In each cell, drug pairs with corresponding side effect and chemical structure similarities are assigned. Then, the average DDI similarities of the drug pairs in the cell are presented in different colors.

In a previous study, the chemical structure and side effect similarities of drugs were shown to be useful for predicting the drug’s target proteins [17]; i.e., drugs with similar chemical structures are likely to target the same proteins, and drugs with similar side effects are also likely to have the same targets. Here, we estimated whether or not similarities determined by DDI can be correlated to similarities obtained by chemical structures and side effects. For this task, we first measured the 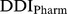 similarity using the shortest path method. The shortest distance between drugs has values between 1 and 6 if there is a path between two drugs, since most drugs are connected within six interactions. Even though 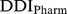 similarity is presented using one of six different values, it can effectively show correlation with other similarities. The correlations between 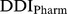 similarity and the chemical structure and side effect similarities are high. The two axes of the grid in Figure 6 represent the side effect and chemical structure similarity, respectively; in the grid, corresponding drug pairs are assigned, and the average DDI similarities of the drug pairs are then presented in a different color. As the side effect and chemical structure similarities increase, the 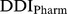 similarity also increases. The Pearson correlation coefficients of 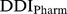-chemical, 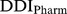-side effect, and chemical-side effect are 0.3005, 0.27, and 0.1896, respectively, thereby confirming that these three features are positively correlated.

#### Integrating DDI Similarity with Chemical Structure and Side Effect Similarities

We first compared the accuracy of each drug-related data source, chemical structure, side effect, and 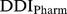, in predicting drug-target interactions. As shown in Table 4, the prediction accuracy of 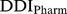 was consistently higher than for the chemical structure and side effects, regardless of the prediction method and drug-target interaction data set. Next, we combined 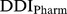 with the chemical structure and side effect data sets, and found that the results of combining these multiple data sets using both SVM and KL1LR were similar to the AUC values obtained by the 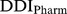 data set. In this research, 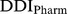 is thus deemed to be the most informative data source.

**Table 4:** Comparison of prediction accuracies of three drug similarities in predicting drug-target interactions.

Methods	DTI	Single drug similarity			Combining multiple drug similarities		
	data source	CH	SE	*DDI_Pharm_*	CS	CSD	CSD (Lanckriet)
KL1LR	ChEMBL	0.7689	0.8109	0.9064	0.7434	0.9055*	–
SVM	ChEMBL	0.8566	0.8947	0.9145	0.9074	0.9382*	0.8342
KL1LR	STITCH	0.7653	0.7653	0.8126*	0.7684	0.8091	–
SVM	STITCH	0.7571	0.7689	0.7937	0.7723	0.7980*	0.7857

AUC values are presented when two prediction methods and two drug-target interaction (DTI) data sets are used. CH and SE indicate the drug similarity based on the chemical structure and side effect, respectively. CS and CSD indicate the drug similarity by combining CH and SE, and combining CH, SE, and DDI, respectively. The last column indicates that the kernel fusion method developed in Lanckriet *et al.* [20] is used for combining multiple kernels in SVM.

* indicates the highest value for each combination of method and data source. For different combinations of methods and data sets, Table S5 contains ROC curves of true positive rate and false positive rate, and tables of true positive, false positive, true negative, and false negative values for each threshold.

In Table 4, KL1LR is seen to be comparable to SVM in terms of its ability to predict drug-target interactions. 13 different 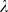 values for KL1LR and 18 different 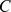 values for SVM were tested as shown in Table S4 (the highest accuracies are shown in Table 4). We also tested various combinations of kernel fusion coefficients of 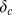, 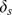, and 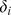 for SVM: (1,1,1), (0.5, 1, 1), (1, 0.5, 1), (1, 1, 0.5), (0.5, 0.2, 1), and (0.8, 0.2, 1). Since all coefficients displayed similar results, we only presented the AUC values when all kernel fusion coefficients were 1. In addition, we tested kernel fusion coefficients generated using the kernel fusion method from [20], [37]; the mean coefficient values of the chemical structure, side effects, and 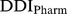 were 0.531, 0.251, and 0.218 for ChEMBL, and 0.414, 0.322, and 0.264 for STITCH, respectively. Accuracies with these coefficients are shown in Table 4, though they were not higher than the case of (1, 1, 1).

**Figure 7:**
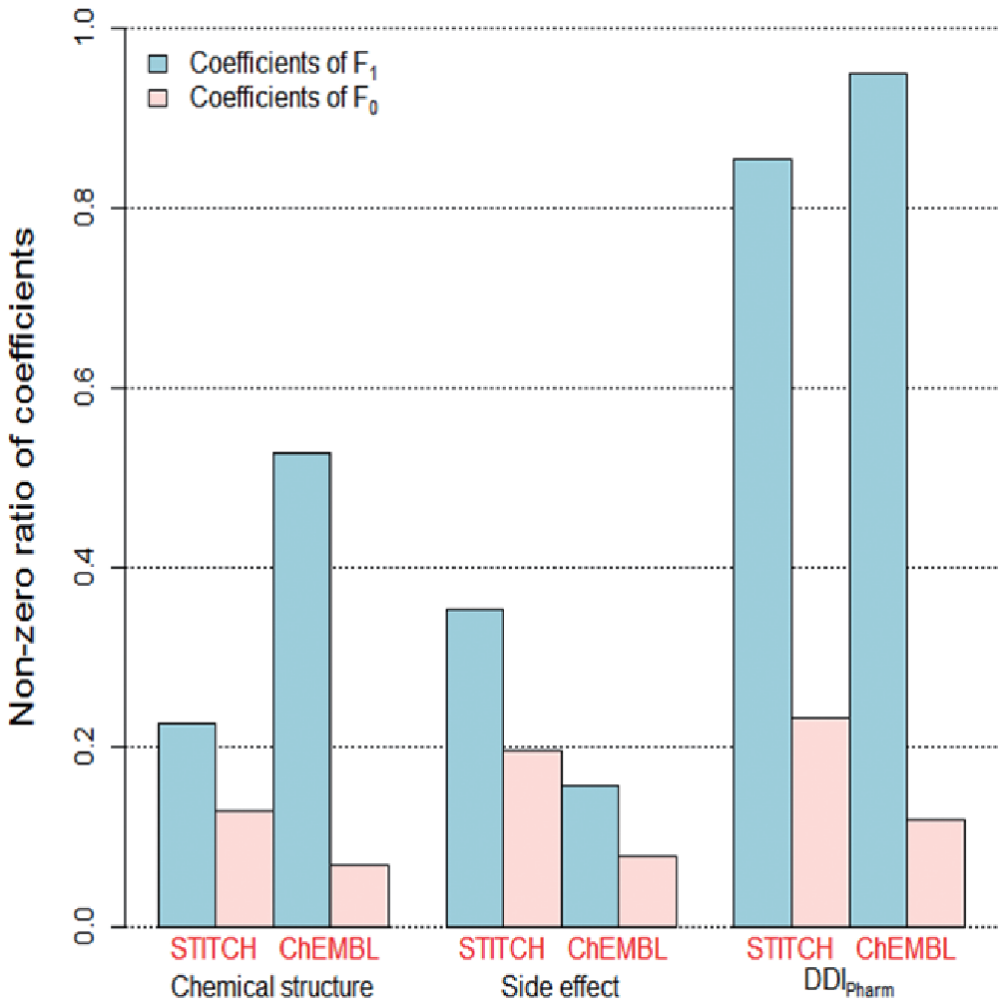
Non-zero ratios of KL1LR coefficients. In Equation (4), 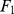 is the average of kernel values between a drug and other drugs that target the given protein; 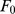 is the average between a drug and other drugs not targeting the given protein. Non-zero ratios are calculated using 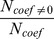, where 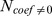 is the number of non-zero coefficients and 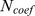 is the number of coefficients.

Since KL1LR assigns unrelated features to zero, we investigated which features have non-zero values. Figure 7 presents the non-zero ratios of coefficients for features from the chemical structure, side effects, and 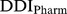. In Equation (4), 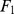 is a feature constructed from the average of similarity values between drug 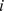 and other drugs that target the given protein. The coefficient of 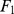 for 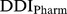 is the highest, which confirms that it contributes the most to the prediction of drug-target interactions.

#### Assessment of positive and negative drug-target interactions

Since STITCH contains interactions from text mining, there is a possibility that some drug-target interactions might be false positives, which in turn might lead to false predictions. To assess how predictions were affected by including interactions from text mining, we performed predictions using only experimental drug-target interactions; the number of drugs, proteins, and drug-target interactions were 261, 2,140, and 4,774, respectively. Based on SVM, the AUC values for the 5-fold cross validation using the chemical structure, side effect, and 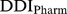 similarities were 0.8149, 0.8360, and 0.8174. When the same 261 drugs and 2,140 proteins were used, with 16,345 drug-target interactions from experiments, text mining, and other databases included for the prediction, the accuracies became 0.7682, 0.7820, and 0.8009 for three different drug similarities, as shown in Table 5; although the performance in this case was decreased compared to the case using only experimental interactions, the difference in performances was not significant. For the KL1LR model, the accuracies were similar for both cases. As such, this comparison shows that our prediction model is reliable even though some false positive interactions were included in the data set.

**Table 5:** Comparison of drug-target interactions predictions from experiments and those from experiments and text mining in STITCH.

Method	Source of drug-target interaction	Chemical structure	Side effect	DDI_Pharm_	CS	CSD
KL1LR	Experiments only	0.7800	0.7744	0.8311	0.8221	0.8288
KL1LR	All	0.7787	0.7768	0.8294	0.7774	0.8267
SVM	Experiments only	0.8149	0.8360	0.8174	0.8410	0.8461
SVM	All	0.7682	0.7820	0.8009	0.7843	0.8078

AUC values for predicting drug-target interactions are shown to compare two cases of using experimentally validated interactions and by using all interactions including experiments, text mining, and other databases. The comparison was conducted using two prediction models (KL1LR and SVM) and three drug similarities (chemical structure, side effect, and 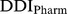) and combining them. CS and CSD indicate the drug similarity by combining CH and SE, and combining CH, SE, and DDI, respectively. For the choice of parameter values, see Table S6.

### Integrating Drug Similarity with Protein Similarity

To this point, we have focused solely on drug similarity. However, since protein similarity could also be a useful resource for predicting drug-target interactions, we further investigated how the prediction accuracy could be increased if the drug and protein similarities are integrated. Prior to this task, we concurrently compared our approach to existing methods [14]–[16]. In the methods described by Bleakley *et al.* [14] and Faulon *et al.* [16], a protein kernel is constructed using the Smith-Waterman alignment of protein sequences. In Jacob *et al.* [15], the protein kernel is constructed using EC numbers. In this comparison, we reduced the ChEMBL and STITCH proteins from 835 and 8,599 to 536 and 1,940, respectively, as some proteins did not have EC numbers. For all three methods, the chemical structure was used to construct the drug kernel: the Tanimoto similarity for Bleakley *et al.* [14] and Jacob *et al.* [15], and the signature kernel for Faulon *et al.* [16], with the height of the signature kernel *h* = 1. Then, to integrate the drug and protein kernels, Bleakley *et al.* [14] used the bipartite local model, and Jacob *et al.* [15] and Faulon *et al.* [16] used the tensor product of two kernels. Here, the computational cost for obtaining the tensor product was very high. For example, the tensor product of 324 compounds and 1,940 proteins produced a (324×1,940) by (324×1,940) kernel size, requiring huge memory resources. To resolve this computational issue, we divided proteins into smaller groups of 15 proteins and then computed the tensor product between 324 compounds and 15 proteins.

For KL1LR, we generated a protein kernel using the Smith-Waterman alignment of protein sequences and then used the bipartite local model to integrate the two probabilities from the drug and protein kernels. To investigate the effect of integrating the kernels, we first measured the accuracy of the drug-target interaction prediction when the chemical structure was used. Table 6 compares the three methods and KL1LR; for comparative purposes, the accuracy of KL1LR with no protein kernel is also shown, confirming that integration with a protein kernel improved the prediction accuracy. The prediction accuracies of the two methods using the bipartite local model (Bleakley *et al.* [14] and KL1LR) are seen to be higher than those using the tensor products (Jacob *et al.* [15], Faulon *et al.* [16]). Next, we used DDI instead of the chemical structure to integrate the drug and protein kernels. From the table, the DDI similarity outperformed the chemical structure similarity regardless of data source and method, thereby confirming the importance of DDI in predicting drug-target interactions. This comparison also shows that the SVM used in Bleakley *et al.* [14] and KL1LR have similar performances when the same bipartite local model is used to integrate the drug and protein similarities.

**Table 6:** Integrating protein similarity and drug similarity.

	Data Source	KL1LR	KL1LR	Bleakley	Jacob	Faulon
		w/o *K_T_*	w *K_T_*			
*K_C_*\*K_T_*		–	Protein	Protein	ECN	Protein
			seq. (*K_l_*)	seq. (*K_h_*)	(*K_l_*)	seq. (*K_l_*)
Ch. struc. (*K_c_*, *K_m_*)	ChEMBL	0.7396	0.9274	0.9557	0.6290	0.6721
Ch. struc. (*K_c_*, *K_m_*)	STITCH	0.7700	0.8535	0.8377	0.6890	0.6847
DDI	ChEMBL	0.9232	0.9290	0.9624	0.7683	0.7678
DDI	STITCH	0.8228	0.8531	0.8649	0.7678	0.6985

Drug-target interaction prediction accuracies using three methods and KL1LR are presented. For KL1LR, two cases of with and without protein similarity are presented. The first column and second row represent the protein similarity and drug similarity used in each method, respectively. Both ChEMBL and STITCH are used as data sources, as shown in the second column. The height of the signature kernel is 1. 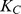 and 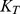 are the drug and protein kernels, respectively. ECN denotes the EC numbers. For the choice of parameter values, see Table S7.

## Discussion and Conclusion

Data sources are one of the most important factors in predicting drug-target interactions. Recently, though several drug and protein related data sources have become available, the range of possible data sources is diverse; the numbers of drugs and proteins are different, and drug-target interactions are not consistent since some interactions might not be contained in the one data set, or other data sets contain false interactions. In addition, since the identifiers for drugs and proteins are different, it is difficult to integrate sets of multiple data sources. In this research, we constructed common sets between ChEMBL and STITCH by using properties of drugs obtained from SIDER side effects, as well as 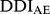 and 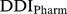 data sets.

Another issue is that STITCH contains interactions from text mining as well as direct chemical-protein binding data, some of which might be false interactions. As shown in the Results section, the differences in performance in terms of predicting drug-target interactions was not significant between two cases in which interactions from text mining were included and excluded. Hence, even though STITCH may contain some indirect drug-target interactions, we included all STITCH interactions to increase the number of potential positive interactions. Also, the observation that our predictions were consistent between ChEMBL and STITCH confirms that our model is reliable for overcoming problems associated with false positive interactions.

To further investigate the relationship between 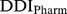 and other drug-related data sets pertaining to chemical structure and side effects, we compared 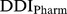, chemical structure, and side effect similarities. It was found that the prediction accuracy of 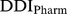 often outperformed the other data sets, and that integration with other data sets improved the prediction accuracy. This result indicates that 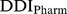 is a primary informative data set for predicting drug-target interactions. One of the advantages of 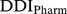 is that the number of compounds having 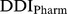 is large; Table 1 shows that 47,911 compounds from STITCH have 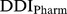 information. Therefore, it is possible that these compounds have potential target proteins, based on the above computational predictions. In this study, however, for comparative purposes, we only used a subset of the compounds that were common in other data sets.

In conclusion, we investigated the significance of DDI for predicting drug-target interactions. Our results showed that 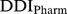 is indeed a useful resource when compared to data sources such as the chemical structures of drugs, drug side effects, and protein sequences. Also, when we used the SVM and KL1LR methods in predicting drug-target interactions, KL1LR was found to be comparable to SVM and useful for investigating the contributing features when several features were integrated.

## Supporting Information

Table S1 **Prediction accuracies with different parameters for** **Table 2** **, where comparing DDIPharm and KEGG pharmacological information.**
(XLSX)

Table S2 **ROC curves and tables of true positives, false positives, true negatives, and false negatives for** **Table 2** **.**
(XLSX)

Table S3 **List of highly ranked unknown drug-target interactions in STITCH.**
(XLSX)

Table S4 **Prediction accuracies with different parameters for** **Table 4** **, where comparing three drug similarities in predicting drug target interactions.**
(XLSX)

Table S5 **ROC curves and tables of true positives, false positives, true negatives, and false negatives for** **Table 4** **.**
(XLSX)

Table S6 **Prediction accuracies with different parameters for** **Table 5** **, where comparing prediction accuracies of drug-target interaction from experiments and those from experiments and text mining in STITCH.**
(XLSX)

Table S7 **Prediction accuracies with different parameters for** **Table 6** **when integrating protein similarity and drug similarity.**
(XLSX)
